# Monkeypox infection in kidney transplant recipients

**DOI:** 10.1093/ckj/sfaf048

**Published:** 2025-02-12

**Authors:** Ola Suliman, Mohammad Joomye, Henry H L Wu, Rajkumar Chinnadurai

**Affiliations:** Department of Renal Medicine, Northern Care Alliance NHS Foundation Trust, Salford, UK; Department of Renal Medicine, Northern Care Alliance NHS Foundation Trust, Salford, UK; Renal Research, Kolling Institute of Medical Research, Royal North Shore Hospital and The University of Sydney, Sydney, NSW, Australia; Department of Renal Medicine, Northern Care Alliance NHS Foundation Trust, Salford, UK; Faculty of Biology, Medicine and Health, The University of Manchester, Manchester, UK

To the Editor,

The monkeypox (Mpox) virus, a double-stranded deoxyribonucleic acid virus belonging to the Poxviridae family of the *Orthopoxvirus* genus, can be transmitted from animals to humans through direct contact with infected animals or their body fluids [1, 2]. Human-to-human transmission occurs via respiratory droplets, contact with bodily fluids, or contaminated materials [1, 2]. Historically, Mpox has been confined to Central and West Africa. However, recent outbreaks have expanded the virus geographically, with cases now being reported worldwide [1]. Whilst there is recognition of the increased risk of Mpox infection in kidney transplant recipients due to their immunocompromised status, there remains limited discussion detailing the disease course and outcomes, and effectiveness of current management strategies for kidney transplant recipients diagnosed with Mpox infection. To this end, we performed a literature search to evaluate currently published clinical cases that described Mpox infection in kidney transplant recipients.

There were 12 full-text case-based publications (11 case reports and 1 case series) published in English between August 2014 and August 2024 that described clinical presentations of Mpox infection in kidney transplant recipients (Table 1) [3–14]. Of these 12 publications, there were 11 Mpox infection cases involving a male patient, where the median age was 43 years [3–14]. Predominant clinical signs and symptoms of Mpox infection for kidney transplant recipients include a widespread skin rash, mucous ulcers, diarrhoea and systemic illness (i.e. fever, fatigue, headache etc.). Descriptions of the nature of skin rash were observed to display variations across the cases—from maculopapular [5] and papulovesicular [3, 10–12] to nodular rashes [8]. Mucosal and oropharyngeal involvement in the form of ulcers was reported in a number of cases [3, 6, 8–10, 12]. There were also other specific symptoms relating to bladder and bowel involvement reported in the published cases—including dysuria, difficulties with bladder emptying [3, 4, 11], and rectal pain [4, 10, 13, 14]. Resolution of rash and symptoms occurred in most cases with the time taken for recovery ranging between 7 days and 4 months. Fuller *et al*. [8] described a patient who eventually developed encephalopathy and multiorgan failure, requiring intensive care admission, where he passed away. This was one of two cases of mortality relating to Mpox infection in kidney transplant recipients that were reported (the other being from the multicentre case series reported by Higgins *et al*. [14], in which the patient developed pneumonitis and circulatory shock and died on day 3 of hospital admission). A meta-analysis by Azzam *et al*. [15] concluded that evidence supporting poor clinical outcomes (i.e. mortality) in individuals with non-human immunodeficiency virus-associated immunosuppression are currently scarce. There were no reported cases of acute kidney injury associated with Mpox infection in kidney transplant recipients. A review article by Reis *et al*. [16] provided recommendations on patient assessment and management in this scenario.

**Table 1:** Summary of published cases describing monkeypox infection in kidney transplant recipients.

Authors	Study design	Age	Gender	Organ transplanted (primary disease if known)	Clinical features/key findings	Management strategy	Clinical outcomes
Attieh *et al*., 2023 [3]	Case report	44	Male	Kidney (HIV nephropathy)	Diarrhoea, oropharyngeal ulcers, papulovesicular rash over entire body, painful urination and difficulty in bladder emptying, fatigue, headache, chills and nausea.	Immunosuppression modification; supportive care; tecovirimat.	Resolution of rash and symptoms in 7 days.
Schmalzle *et al*., 2023 [4]	Case report	62	Male	Kidney (HIV nephropathy)	Fatigue, chills, rectal pain, dyspnoea and skin lesions.	Immunosuppression modification; tecovirimat.	Resolution of rash from 14 to 41 days.
Alalawi *et al*., 2023 [5]	Case report	46	Male	Kidney (FSGS)	Fever, sore throat, diarrhoea and maculopapular rash in upper body.	Empirical antibiotics; immunosuppression modification.	Resolution of rash in 3 weeks.
Colina-García *et al.*, 2024 [6]	Case report	43	Female	Kidney (diabetic nephropathy)	Skin and mucosal ulcers and lung opacities.	Empirical antibiotics; immunosuppression modification; tecovirimat; IgG; cidofovir	Prolonged illness (>2 months) with residual respiratory issues.
Rousseau *et al.*, 2023 [7]	Case report	58	Male	Kidney-pancreas (diabetic nephropathy)	Skin rash and fever.	Tecovirimat; no change in immunosuppressive therapy	Complete healing 4 months.
Fuller *et al.*, 2023 [8]	Case report	53	Male	Kidney (hypertensive nephropathy)	Flank pain, haematemesis, nodular skin rash and mucosal lesions, encephalopathy and multiorgan failure.	ICU support; empirical antibiotics; tecovirimat; cidofovir.	Fatal outcome.
Caillard *et al.*, 2023 [9]	Case report	25	Male	Kidney (nephronophthisis)	Skin and mucosal lesions.	Tecovirimat; immunosuppression modification.	Gradual resolution of lesions.
Alameer *et al.*, 2024 [10]	Case reports x 2	30	Male	Kidney	Widespread papulovesicular skin rash and oral ulcers, rectal pain, fever, headache and fatigue.	Acyclovir; empirical antibiotics; immunosuppression modification; brincidofovir.	Successful recovery in both patients, with no significant side effects observed from brincidofovir.
		25	Male	Kidney (FSGS)	Widespread vesicular skin rash and rectal pain		
Saleh *et al.*, 2023 [11]	Case report	44	Male	Kidney (HIV nephropathy)	Diarrhoea, dysuria, urinary retention and vesicular skin lesions.	Tecovirimat; empirical antibiotics; immunosuppression modification.	Resolution of symptoms 8 days.
Heis *et al.*, 2023 [12]	Case report	30	Male	Kidney (FSGS)	Diffuse vesicular lesions and mucosal involvement and fever.	Tecovirimat; supportive care.	Resolution of symptoms in 4 weeks.
Menéndez Garcia *et al.*, 2023 [13]	Case report	26	Male	Kidney (aHUS)	Fever, diarrhoea, papular lesions in anal region and rectal pain and bleeding.	Tecovirimat; supportive care.	Resolution of symptoms in 1 week.
Higgins *et al.*, 2023 [14]	Multicenter case series with a literature review.	Not specified	All males	91% of patients had kidney transplantation, the other 9% had various organs transplanted	Fever, fatigue, proctitis, and pharyngitis.Severe cases showed complications like pneumonitis and circulatory shock.	All patients received tecovirimat, supportive care.	Resolution of symptoms in all but one patient by 30-day follow-up. Hospitalization rate was 73%, with a median stay of 4.5 days. One mortality was linked to Mpox infection.

aHUS, atypical haemolytic uraemic syndrome; FSGS, focal segmental glomerulosclerosis; HIV, human immunodeficiency virus; ICU, intensive care unit; IgG, immunoglobulin G.

For the majority of the reported cases, standard management included supportive care with modification of immunosuppression if indicated (i.e. suspension of antiproliferative agents such as mycophenolate mofetil or azathioprine; administering tacrolimus at lower levels) and prescription of tecovirimat, originally developed for the treatment of smallpox and the first antipoxviral medication approved in the USA [3, 4, 7, 9, 11–14]. These management measures alone led to the resolution of rash and symptoms in most of the cases. Alameer *et al*. [10] described the prescription of brincidofovir, which is not a routinely available antiviral option for Mpox. No significant side effects from brincidofovir were observed nevertheless. Antibiotics were administered in several of the cases, either empirically whilst awaiting confirmation for any bacterial sources or due to infective complications with detection of bacterial organisms. Empirical antibiotic initiation was observed in the cases by Alalawi *et al*. [5] (antibiotic not specified), Fuller *et al*. [8] (vancomycin and cefepime) and Alameer *et al*. [10] (piperacillin/tazobactam and doxycycline were commenced for both cases). In the case reported by Colina-Garcia *et al*. [6], the patient was superinfected with multidrug-resistant *Pseudomonas aeruginosa* at the end of their admission and treated with aztreonam. Whilst there was stabilized recovery eventually despite prolonged illness of >2 months, the patient experienced residual respiratory issues post-discharge. The case patient from Saleh *et al*. [11] developed stool culture positivity for enteropathogenic *Escherichia coli* and he was treated with intravenous ceftriaxone during admission.

In summary, currently reported cases underscore the need for vigilant monitoring and identification of Mpox infection in kidney transplant recipients. Timely initiation of supportive care and tecovirimat administration when indicated has led to patient recovery in most of the reported cases. Continued study is required to develop tailored guidance for managing kidney transplant recipients with Mpox infection.
